# Optimized Method to Generate Well-Characterized Macrophages from Induced Pluripotent Stem Cells

**DOI:** 10.3390/biomedicines13010099

**Published:** 2025-01-03

**Authors:** Qimin Hai, Peter Bazeley, Juying Han, Gregory Brubaker, Jennifer Powers, Claudia M. Diaz-Montero, Jonathan D. Smith

**Affiliations:** 1 Department of Cardiovascular & Metabolic Sciences, Lerner Research Institute, Cleveland Clinic, Cleveland, OH 44195, USA; 2 Department of Quantitative Health Sciences, Lerner Research Institute, Cleveland Clinic, Cleveland, OH 44195, USA; 3 Immunomonitoring Laboratory, Center for Immunotherapy and Precision Immuno-Oncology, Lerner Research Institute, Cleveland Clinic, Cleveland, OH 44195, USA; 4 Department of Molecular Medicine, Cleveland Clinic Lerner College of Medicine of Case Western Reserve University School of Medicine, Cleveland, OH 44195, USA

**Keywords:** iPSC, macrophage, cell differentiation, embryoid body, polarization, M1-like macrophage, M2-like macrophage, transcriptome

## Abstract

**Background/Objectives**: Macrophages play a pivotal role in various pathogenic processes, necessitating the development of efficient differentiation techniques to meet the high demand for these cells in research and therapy. Human macrophages can be obtained via culturing peripheral blood monocytes; however, this source has limited yields and requires patient contact for each proposed use. In addition, it would be difficult to perform gene editing on peripheral blood monocytes. The objectives of this study are to define a robust and consistent method for the differentiation of induced pluripotent stem cells (iPSCs) into macrophages that can address these needs for recurrent studies with high yields and the potential for gene editing. **Methods**: We refined the traditional embryoid body-based differentiation strategy to create a novel three-phase method that optimizes yield, consistent quality, and reproducibility. This approach incorporates microwell plates and cell filtration to standardize the production of embryoid bodies and subsequent macrophage progenitors. Using up to five independent iPSC donors, we performed several assays for macrophage functions and polarization, such as marker protein staining by flow cytometry, lipoprotein uptake, phagocytosis, cytokine release, inflammasome activation, and the effects of M1-like and M2-like polarization. RNA sequencing was performed to determine the segregation of cells at different stages of differentiation and by iPSC donor, as well as to identify marker genes for each stage of differentiation. **Results**: The iPSC-derived macrophages generated through this method exhibit characteristic features and cell marker proteins, as well as classical macrophage activities, including lipoprotein uptake, bacterial phagocytosis, cytokine release, and inflammasome activation. We demonstrate the effects of M1-like and M2-like polarization on cytokine release. The first three principal components of the RNA sequencing data showed clear clustering by differentiation stage. In contrast, the fourth and fifth principal components clustered the differentiated macrophages by their respective iPSC donor. Marker genes were identified for each stage of differentiation and polarization. **Conclusions**: The methods provide an optimized and simplified procedure to produce iPSC-derived macrophages. Our results demonstrate the reproducibility of this method in generating high-quality macrophages suitable for a variety of biomedical applications.

## 1. Introduction

Macrophages play vital roles in a myriad of pathogenic and physiological processes, making them highly sought after in biomedical research. Macrophages are derived from two sources: hematopoietic monocytes that yield patrolling macrophages and yolk sac-derived that yield tissue macrophages such as Kupffer cells [1,2]. Mouse macrophages can be readily prepared from peritoneal lavage or bone marrow differentiation, but these are not feasible sources for human macrophages. Instead, peripheral blood monocytes, upon culture for 3–7 days, have been used as a source of human macrophages for laboratory use [3]. However, even this source has its limitations regarding cell yield and the inability to regenerate the monocytes, such that when all the monocytes have been used in experiments, one must recontact the donor and obtain additional blood. A reliable and steady source of human macrophages is required for mechanistic, genetic, and synthetic biology purposes. Since the discovery of the Yamanaka factors to produce human induced pluripotent stem cells (iPSCs) [4], numerous methods have been developed to produce many types of differentiated cells. The rationale for this study is that the differentiation of induced pluripotent stem cells (iPSCs) into macrophages can meet the need for recurrent studies with high yields and the potential for gene editing. Existing protocols for macrophage differentiation from iPSCs can be divided into two major categories: monolayer-based and embryoid body-based methods.

The monolayer protocol entails a complex procedure involving mesoderm formation, hemogenic endothelium development, hematopoietic induction, and myeloid specification using multiple exogenous growth factors [5]. Efforts to simplify macrophage differentiation have led to adopting the embryoid body (EB)-dependent approach [6,7,8,9], which reduces procedural complexity. However, this method introduces significant variability, as iPSCs aggregate randomly during differentiation to form EBs of varying sizes, thereby introducing inconsistency that can impede reproducibility. Recognizing the need for a more reliable approach, this study introduces a standardized methodology for the differentiation of iPSCs into macrophages. Our method comprises three phases: (1) the formation of EBs; (2) the generation of macrophage progenitors; and (3) the final maturation into macrophages. This streamlined method aims to consistently produce standardized human iPSC-derived macrophages (iPSMs), optimizing the yield and quality necessary for advanced studies. Here, we provide our detailed step-by-step differentiation protocol with immunological and functional characterization of the resultant iPSMs.

## 2. Materials and Methods

### 2.1. Detailed Step-by-Step Protocol for Differentiation of iPSMs

**Figure 1:**
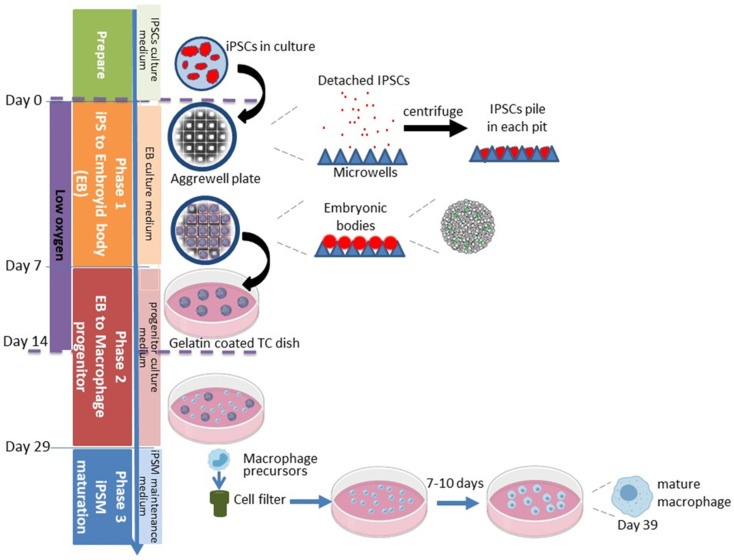
Optimized schematic for iPSC differentiation to macrophages. This method uses a three-phase-based process for the generation of EBs in phase 1, the production and collection of macrophage progenitor cells in phase 2, and the maturation of iPSMs in phase 3.

The general iPS to iPSM procedure in three phases is illustrated in Figure 1.

#### 2.1.1. Human iPSC Culture

- Matrigel-coated plates:

1. Thaw a tube of growth factor-reduced Matrigel (Corning, Corning, NY, USA # 354230) on ice;
2. Add 120 µL Matrigel to 12 mL of DMEM/F12 (Thermo Fisher, Waltham, MA, USA # 12500096) at 4 °C (enough for 1–2 plates) and put it in ice;
3. Plate 0.5 mL of the mix per well in two 12-well plates (Falcon Corning, Corning, NY, USA # 353043, or equivalent).
4. Place plates in a 37 °C incubator for at least 30 min. Plates may be kept for up to 2 weeks in a 37 °C incubator without drying out.

- Thawing and initial plating of iPSCs:

1. The following iPS cell lines were obtained from the CIRM hPSC Repository funded by the California Institute of Regenerative Medicine (CIRM, South San Francisco, CA, USA): CW50067BB1, CW50009BB1, CW20105DD1, CW50075AA1, and CW30391CC1. All donors consented to research using their cell lines; the donor consent forms followed guidelines set by CIRM and were approved by Institutional Review Boards (IRBs). All the materials were collected under IRB from the Institutions that collected the material;
2. Thaw the frozen iPS vial in a 37 °C water bath until just thawed. Add thawed cells to 5 mL of mTeSR Plus medium (Stemcell Technologies, Vancouver, BC, Canada # 100-0276) in a 15 mL conical tube (Falcon Corning, Corning, NY, USA # 352097). Spin and wash cells with ~1 mL of mTeSR Plus medium. Spin cells and resuspend in 2 mL of mTeSR Plus medium containing 10 uM Y2763 ROCK inhibitor (Tocris, Minneapolis, MN, USA # 1254). Transfer 1 mL of cells each into two wells of the Matrigel-coated 12-well plate. Place into a hypoxic (5% O_2_) incubator;
3. Change media every day with 1 mL of mTeSR+ medium (*w*/*o* Rock inhibitor).

- Passage of iPSCs by the clump culture method:

1. Ideally, cells should be growing in small clumps and reach 65–80% confluence before passage. Split at 1:3 to 1:5 every 3–4 days to avoid confluence which can lead to spontaneous loss of pluripotency;
2. Rinse cells w/PBS, add 300 μL Accutase (Gibco, Waltham, MA, USA # A1110501), and incubate for 5 min at 37 °C. With a P1000 tip, add 1 mL of mTeSR+ w/ROCK inhibitor, and gently pipet up and down to lift cells from the plate in small clumps without dissociating into single cells. Dilute cells as needed in mTeSR+ w/ROCK inhibitor and plate cells into 3–5 wells of a Matrigel-coated 12-well plate;
3. Change media every day with 1 mL of mTeSR+ medium (*w*/*o* Rock inhibitor).

- Cryopreservation of iPSCs:

1. Rinse cells w/PBS, add 300 μL Accutase, and incubate for 5 min at 37 °C. With a P1000 tip, add 1 mL of mTeSR+ w/ROCK inhibitor, and gently pipet up and down to lift cells from the plate in small clumps without dissociating into single cells;
2. Spin down the cells, resuspend w/1 mL of mFreSR medium (Stemcell Technologies, Vancouver, BC, Canada # 05855) containing 10 μM Rock inhibitor;
3. Transfer 1 mL of cells to cryovial. Transfer the vial to a slow freezing container and store at −80 °C overnight, then transfer the frozen tubes to a liquid nitrogen tank for long-term storage.

#### 2.1.2. Phase 1: Embryoid Body Formation

**Figure 2:**
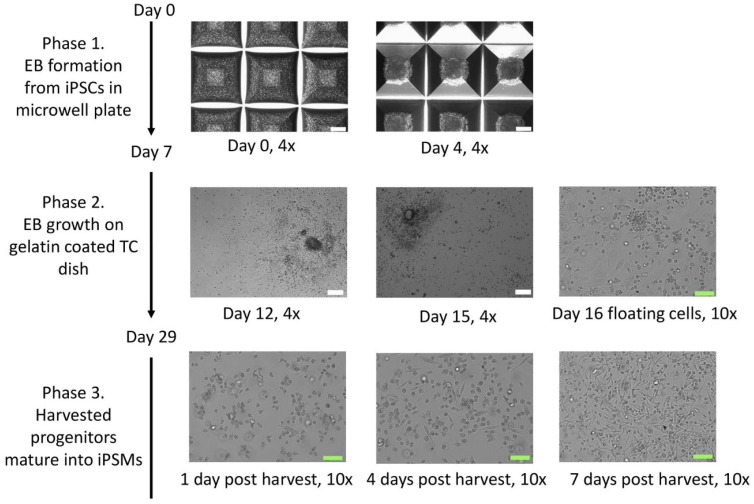
Bright-field microscopy of macrophage differentiation during phases 1–3. The time points are as depicted, and images were obtained using a 4× or 10× objective lens with a white scale bar of 200 microns and a green scale bar of 100 microns.

- Growth of iPSCs to not more than 50% confluence on a Matrigel-coated P100 TC dish:

1. When iPSCs reach 65–80% confluence in a well of a 12-well plate, detach iPSC with Accutase as above, but pipet cells thoroughly to obtain single-cell suspension;
2. Count cells and plate 7 × 10^5^ cells onto a Matrigel-coated P100 plate (Corning, Corning, NY, USA # 353003) as described above;
3. Change to mTeSR+ medium *w*/*o* Rock inhibitor the next day;
4. About 2 days later the iPSC should not be higher than 50% confluent. Keeping the cells at this density is a critical step for successful iPSM differentiation.

- Formation of embryoid bodies in AggreWell microwell plate:

1. Pre-treat one well for each iPS line in a 24-well AggreWell 800 plate (each well has 300 micropits) with a 500 μL Anti-Adherence Rinsing Solution (both contained in Stemcell Technologies, Vancouver, BC, Canada # 34850);
2. Centrifuge plate at 1300× *g* for 5 min in a swinging bucket rotor fitted with plate holders. Observe the plate under a microscope to ensure bubbles have been removed from microwells. If bubbles remain trapped in any microwells, centrifuge at 1300× *g* for an additional 5 min;
3. Aspirate Anti-Adherence Rinsing Solution from the wells. Rinse each well with 2 mL of warm mTeSR Plus medium;
4. Aspirate medium from the well. Add 1 mL of warm EB medium (Table 1) supplemented w/Rock inhibitor to each well to be used;
5. Rinse 50% confluent iPSC w/PBS and detach iPSCs with Accutase, as described above, but pipet thoroughly into single cells. Then add mTeSR Plus to quench Accutase, as shown above;
6. Count cells, spin down 4.5 × 10^6^ IPSCs at 250× *g* for 5 min. Resuspend pellet in 1 mL of EB medium supplemented w/Rock inhibitor (10 μM);
7. Add cells to well to yield a final volume of 2 mL;
8. Pipette cells up and down gently several times with a P1000 pipettor to ensure even distribution. Spin plate at 100× *g* for 3 min. Under the microscope, piled iPSCs should be observed in each microwell (Figure 1 and Figure 2);
9. Incubate the plate at 37 °C in a 5% O_2_ 5% CO_2_ incubator. Feed with 1 mL of EB medium (*w*/*o* rock inhibitor) every day for 7 days. Compact EB should be observed by day 2 (Figure 2).

#### 2.1.3. Phase 2: Generation of Macrophage Progenitors

1. Add 10 mL of macrophage progenitor culture medium (Table 2) to gelatin-coated (4 mL of 0.1% gelatin incubated at 37 °C for 1 h, MP, catalog number: 901771) P100 tissue culture dish;
2. Remove and discard 0.5 mL of media from each well of the EB culture, yielding 1.5 mL remaining. Cut a P1000 pipet tip with a sterile blade to widen the opening. Gently suspend EBs with a P1000 pipet tip to dislocate each EB from the microwells;
3. Transfer free EBs to a 1.5 mL sterile microfuge tube. EBs will spontaneously sediment in 1 min. Discard the supernatant and use wide-open P1000 pipet tip to resuspend the EBs with progenitor culture medium;
4. Transfer resuspended EBs into the previously prepared P100 tissue culture dish mentioned above;
5. Move the P100 dish back-and-forth and side-to-side, without rotating, to evenly distribute EBs and incubate in a hypoxic incubator for the first 7 days of phase 2, then move to a regular incubator. Replenish with progenitor culture medium every 4 days. EBs will attach to the P100 dish and begin to grow out.

#### 2.1.4. Harvesting Macrophage Progenitors

Over time, macrophage progenitors are released from the EBs and float in the medium. Starting on day 29, floating progenitors in 10 mL of medium may be harvested every 4 days, which are then passed through a sterile 50 µm cell filter (Sysmex, Lincolnshire, IL, USA # 04-004-2326), to remove EB fragments and plated onto a new gelatin-coated P100 tissue culture plate. The phase 2 EBs are replenished with 10 mL of progenitor culture medium.

#### 2.1.5. Phase 3: iPSMs Maturation and Maintenance

Macrophage progenitors attach to the gelatin-coated plates overnight, and the medium is replaced with a macrophage maintenance medium (Table 3). Replenish cells with fresh maintenance medium every 4 days until usage (usually 7–10 days after harvesting progenitors).

#### 2.1.6. Cryopreservation of iPSMs

Freeze down mature iPSM 7 to 10 days after harvesting progenitors. Ideally, cells should have reached 65–85% confluence. Discard the supernatant and rinse once w/PBS. Add 3 mL of Accutase to the phase 3 P100 plate, incubate for 5 min at 37 °C, and gently detach with a sterile cell lifter (Corning, Corning, NY, USA # 3008). Transfer the cell mixture into a 15 mL sterile tube containing 3 mL of maintenance medium. Count the cell number and pellet the cells by spinning for 5 min at 200× *g*. Resuspend the cells at 1 × 10^6^ cells/mL in an iPSM freezing medium (5% DMSO in 95% FBS). Aliquot 1 mL of cells into each cryovial. Transfer the vial to a slow freezing container and store at −80 °C overnight, then transfer the frozen tubes to a liquid nitrogen tank for long-term storage. To reconstitute frozen cells, thaw cells in a 37 °C water bath until liquid, and transfer cells to a 1.5 mL tube containing 0.5 mL of maintenance medium. Pellet and replate cells in maintenance medium at ~110,000 cells per cm^2^ of gelatin-coated plate. Cells are still capable of limited cell division.

### 2.2. Immunological Profiling of iPSCs, Macrophage Progenitors, and iPSMs by Flow Cytometry

iPSCs, macrophage progenitors, and iPSMs were fixed with a Fixation buffer (BioLegend, San Diego, CA, USA # 420801) according to the manufacturer’s instructions. Cells were rinsed with PBS and lifted as described, then pelleted and resuspended in a 0.5 mL Fixation Buffer in the dark for 20 min at room temperature. The fixed cells were pelleted, resuspended with Cell Staining Buffer (BioLegend, San Diego, CA, USA # 420201), and stored at 4 °C. Fresh antibody cocktail was prepared using titrated antibodies listed in Table 4 with 25 μL Horizon Brilliant Stain buffer (BD Biosciences, Franklin Lakes, NJ, USA # 566349) per test. ~1 million cells for each cell sample were incubated with the antibody cocktail for 20 min protected from light at room temperature in flow cytometry (FC) tubes. Samples were washed with 2 ml of FC buffer (PBS with 2% heat-inactivated FBS) and centrifuged at 300× *g* for 6 min. The pellet was resuspended in 250 μL FC buffer and vortexed. Samples were run on a BD Biosciences (Franklin Lakes, NJ, USA) FACSymphony A5 with 5 lasers (349 nm, 402 nm, 488 nm, 532 nm, 628 nm, 30 parameter +FSC and SSC). Prior to data acquisition, the A5 was calibrated using a cytometer setup and tracking beads (BD Biosciences, Franklin Lakes, NJ, USA # 642412), and compensation was set up using UltraComp eBeads Plus (Invitrogen, Waltham, MA, USA # 01-333-42) as directed. Unstained cells were also acquired to account for the high autofluorescence of the macrophage subtypes and used for gating purposes. Analysis was performed with Flow Jo v10.8. Cells were gated on FSC and SSC, and cell doublets were excluded from further analysis.

### 2.3. Acetylated LDL Uptake Assay

Aceteylated LDL (AcLDL) was prepared from blood bank expired human plasma and then labeled with the DiI fluorophore as previously described [10]. 50 µg/mL DiI-AcLDL was incubated with iPSMs in a regular maintenance medium and incubated at 37 °C for 25 min. Cells were washed in PBS and analyzed by epifluorescence microscopy or flow cytometry.

### 2.4. Bacteria Phagocytosis Assay

Mature iPSMs were detached and plated at 50,000 cells/well in a 96-well glass bottom plate. Cells were washed once with PBS+Ca^2+^Mg^2+^ and then incubated in 5 µM of Vybrant CFDA SE Cell Tracer (ThermoFisher, Waltham, MA, USA # V12883) in PBS+Ca^2+^Mg^2+^ at 37 °C for 15 min to stain live cells. After washing, 200 μL of maintenance medium was added to the cells. Then, 20 μL of 1:1000 diluted pHrodo red labeled *E. coli* Bioparticles (InVitrogen, Waltham, MA, USA # P35361) was added and incubated for 30 min at 37 °C. Cells were washed twice in PBS+Ca^2+^Mg^2+^ and imaged by epifluorescence microscopy.

### 2.5. Cholesterol Efflux Assay

iPSMs were detached and plated at 250,000 cells/well in 24-well plates. The cells were labeled overnight with 0.5 µCi/mL 3H cholesterol in RPMI supplemented with 100 ng/mL M-CSF and 1% fetal bovine serum. Afterwards, the wells were washed twice with prewarmed PBS. Cholesterol efflux was assessed by 16 h incubation in 0.5 mL of RPMI with 100 ng/mL M-CSF in the presence or absence of 5 µg/mL recombinant human apoA1, prepared as previously described [11]. The 3H dpm in the media spun to remove cell debris and extracted from the cells in hexane:isopropanol (3:2) was determined by liquid scintillation counting. % Cholesterol efflux was calculated as 100 × dpm in medium/(dpm media + cells).

### 2.6. Polarization of iPSMs

To obtain M1-like macrophages, ~10 days old iPSMs were incubated for 24 h in a maintenance medium containing low-dose LPS (100 ng/mL) and INFγ (20 ng/mL). To obtain M2-like macrophages, ~10-day-old iPSMs were incubated for 24 h with IL4 (20 ng/mL) in a maintenance medium.

### 2.7. Cytokine Release Assay

iPSM were left untreated (M0) or polarized to M1-like or M2-like cells for 24 h. The media was collected, spun to remove cell debris and used fresh or stored at −80 °C. Multiple cytokines were measured simultaneously by flow cytometry using the LEGENDplex Human M1/M2 Macrophage Panel (BioLegend, San Diego, CA, USA # 740509) according to the manufacturer’s protocol.

### 2.8. Inflammasome Activation and IL-1β Secretion Assay

iPSMs were first primed with a high dose (1 μg/mL) of LPS from Escherichia coli O55:B5 (Sigma, Burlington, MA, USA # L6529) for 4 h at 37 °C. This was followed by a treatment with 5 mM adenosine triphosphate (ATP) (Sigma; A2383) for 30 min. After treatment, the media were collected and briefly centrifuged to remove cellular debris. The levels of IL-1β in the supernatants were quantified using a human IL-1β ELISA kit according to the manufacturer’s instructions (R&D Systems, Minneapolis, MN, USA # DLB50). To normalize the released IL-1β levels, cellular protein content was determined using the bicinchoninic acid (BCA) protein assay (ThermoFisher, Waltham, MA, USA # 23227) on total cell lysates, which were prepared by incubating the cells for 4 h at 37 °C in a solution of 0.2 N NaOH and 0.2% SDS.

### 2.9. RNAseq Analysis

iPS cells, macrophage progenitors, M0, M1-like, and M2-like macrophages were lysed directly in trizol buffer for RNA extraction. RNA was purified using miRNeasy Kit (Qiagen, Germantown MD, USA # 217004) according to the manufacturer’s protocol. Oligo-dT cDNA libraries were prepared and paired end 100 bp sequencing was performed on a NovaSEQ-X instrument at the University of Chicago Genomics Core. The nf-core/rnaseq [12] (revision 3.14.0) pipeline was used to trim reads with option “fastp” with additional parameters “extra_fastp_options: ‘--qualified_quality_phred 20 --unqualified_percent_limit 40 --trim_poly_g’”, remove ribosomal reads and align reads to the human genome build GRCh38.p14 from Gencode version 46 with option “star_salmon” with additional parameters “’extra_salmon_quant_args: --seqBias --gcBias’”. Within-donor sample genetic concordance was assessed by identity-by-state calculated with plink [13] (version 1.90b6.21) on RNA variants called by DeepVariant [14] (version 1.4.0) and combined with glnexus [15] (version 1.4.1-0-g68e25e5) based on the protocol described at “https://github.com/google/deepvariant/blob/r1.6.1/docs/deepvariant-rnaseq-case-study.md”, assessed 1 July 2024.

Downstream analyses were performed with R [16] (version 4.3.3). Transcript-level TPM were imported and summarized to gene-level counts with package tximport [17] (version 1.30.0) with option “type = ‘salmon’”. Principal component analysis (PCA) was performed on gene counts centered on 0 after transforming to the log2 scale with function “rlog” from package DESeq2 [18]. Gene counts were additionally summarized with the tximport option “countsFromAbundance = ‘lengthScaledTPM’” and TMM-normalization, gene filtering, and calculation of CPM were performed with package edgeR [19]. Differential gene expression was performed on TMM-normalized counts with the “limma-voom” method from package limma [20], comparing each cell type to the rest while accounting for within-donor correlation. Heatmap of marker genes based on CPM values after replacing 0 values with 0.1, averaging within each cell type and log2-transforming. The RNAseq DESeq2 normalized CPM are available via Gene Expression Omnibus (GEO) accession # GSE277609.

### 2.10. General Statistical Analysis

General statistical analysis was performed using GraphPad Prism version 10.1.2. All data are presented plus or minus standard deviation unless specified otherwise.

## 3. Results

### 3.1. Generation of iPSMs from iPSCs

iPSCs are centrifuged gently in the AggreWell plate, where the cells settle into the micropits such that each micropit has the same number of cells, which facilitates the individual and uniform growth of large, spherical embryoid bodies (EB). The EBs are formed in phase 1 for 7 days in a low-oxygen environment. Figure 2 shows the morphology of EBs in the AggreWell plate on days 0 and 4. We found that keeping the EBs in the low oxygen environment in phase 1 and the first 7 days of phase 2 led to better adherence and growth after the EBs were transferred to phase 2. The appearance of EB growth in phase 2 is shown in Figure 2. The EBs spread concentrically until reaching confluence. By day 16, the EBs begin releasing floating monocyte-like macrophage progenitors, which can be harvested starting day 29. Additional rounds of progenitors can be harvested every 4 days. We found that it was important to filter the progenitors, removing EB fragments and cell aggregates when harvesting the progenitors to initiate phase 3. Otherwise, the synchronized differentiation of iPSMs will be disturbed by the presence of the less mature EBs that continually release progenitors. During phase 3, the progenitors undergo limited division as they elongate and spread out during their transition into terminal macrophages (Figure 2), completing their maturation within 7 to 10 days after plating.

### 3.2. Immunological Characterization of iPSMs and Their Precursors

**Figure 3:**
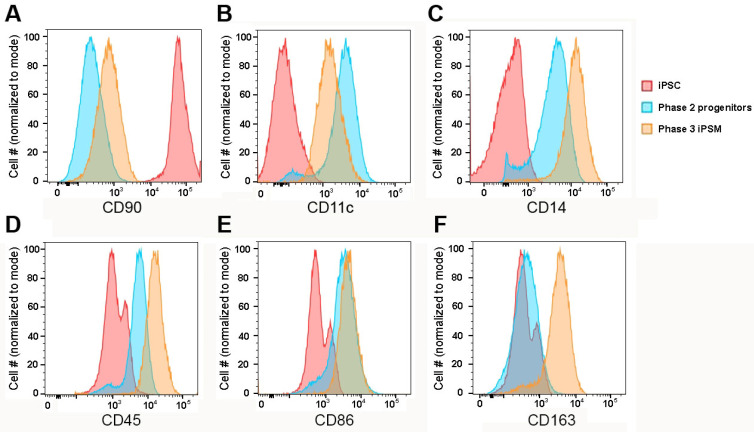
Expression of cell marker antigens in iPSCs, progenitors, and mature iPSMs ascertained by flow cytometry. X-axis, fluorescence intensity; Y-axis, normalized cell counts. (**A**–**F**) CD90, stem cell marker; CD11c, myeloid cell marker; CD14, myeloid marker; CD45 pan leukocyte marker; CD86, myeloid and B-cell marker; and CD163, macrophage-specific marker, respectively. Red, iPSCs; blue, macrophage progenitors; and orange, iPSMs. Forward and side scatter gating for these cells is shown in Supplemental Figure S1.

To confirm the identity of the derived cells, we conducted immunofluorescence staining and flow cytometry analyses on the iPSCs, floating macrophage progenitors, and mature iPSMs. The stem cell marker CD90, encoded by the THY1 gene, was highly expressed in iPSCs; however, its expression was markedly reduced in both progenitors and iPSMs, indicating a loss of pluripotency markers as differentiation progressed (Figure 3A). The expression of the leukocyte-specific integrin αX, CD11c, was negative in iPSCs and increased in both progenitors and iPSMs (Figure 3B). The monocyte lineage-specific marker CD14 and leukocyte common antigen CD45 were not expressed in IPSCs and progressively increased from moderate in progenitors to high in iPSMs (Figure 3C,D). The dendritic cell, memory B cell, and macrophage maker CD86 were not expressed on iPSCs, and moderately and equally expressed on both progenitors and iPSMs (Figure 3E). The macrophage-specific marker CD163 was negative/low on both iPSCs and progenitors and expressed highly on iPSMs (Figure 3F).

### 3.3. Optimal iPSM Differentiation Time Course

**Figure 4:**
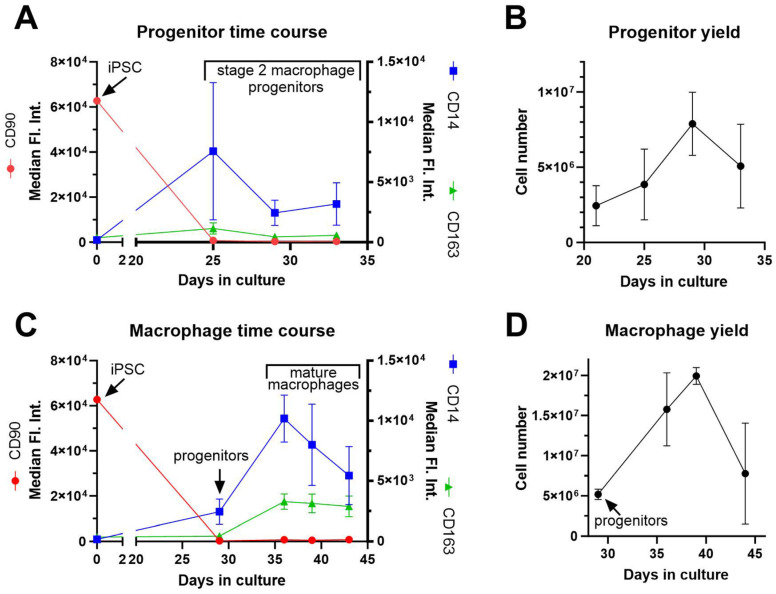
Time course and yield optimization of iPSM differentiation. (**A**) Characterization of phase 2 macrophage progenitors. Five iPSC lines underwent differentiation for expression levels of the CD14 (monocyte cell marker), CD163 (macrophage marker) and CD90 (stem cell marker) at day 0 and 3 time points throughout phase 2 (at days 25, 29, and 33). (**B**) Quantification of phase 2 cell yield at days 21, 25, 29, and 33, using 5 iPSC lines. (**C**) Characterization of phase 3 iPSMs. Three iPSC lines underwent differentiation for expression levels of the CD14, CD163, and CD90 at days 0, 29 (at progenitor harvesting), 36, 39, and 43 (the latter 3 time points throughout phase 3 are 7, 10, and 14 days after progenitor harvesting). (**D**) Time-course of iPSM yield at 4 time points during phase 3 on the same 3 iPSC lines depicted in panel (**C**). All panels display mean + S.D.

Time-course experiments were performed to monitor the quality and quantity of progenitors and iPSMs. Cell differentiation status was assessed by flow cytometry using CD90 (stem cell-specific), CD14 (progenitor and iPSM-specific), and CD163 (iPSM-specific). Five iPSC cell lines from different donors were evaluated along with floating phase 2 progenitors collected at days 25, 29, and 33. CD90 expression was high in iPSCs and was low/negative in progenitors at all three time points. CD14 expression was negative in iPSCs and was expressed moderately in progenitors at all three time points. CD163 expression was negative/low in both iPSCs and all progenitor time points (Figure 4A). The yield of progenitors steadily increased until day 29, after which a decline was observed (Figure 4B). At day 29 we obtained ~8 million progenitors derived from 300 phase 2 EBs.

Phase 2 progenitors harvested at day 29 and derived from three distinct iPSC cell lines were used to examine macrophage maturation and yield at three time points during phase 3. Phase 3 iPSMs were harvested at days 36, 39, and 43 (corresponding to 7, 10, and 14 days after plating progenitors). CD14 expression was higher in iPSMs vs. progenitors and peaked at day 36. CD163 expression was negative/low in progenitors and remained consistently high in iPSMs at days 36, 39, and 43. CD90 expression remained low/negative throughout the iPSM time course (Figure 4C). The yield of iPSMs peaked at day 39, 10 days after plating progenitors, yielding ~20 million macrophages (Figure 4D).

It is also possible to generate iPSMs from progenitors harvested on days 33 and thereafter. In this case, the time course for iPSM maturation for each plating is based on the days after progenitor plating. However, progenitor yield peaks at day 29, and we prefer to use only the first two progenitor harvests on days 29 and 33.

### 3.4. Functional Characterization of iPSMs

**Figure 5:**
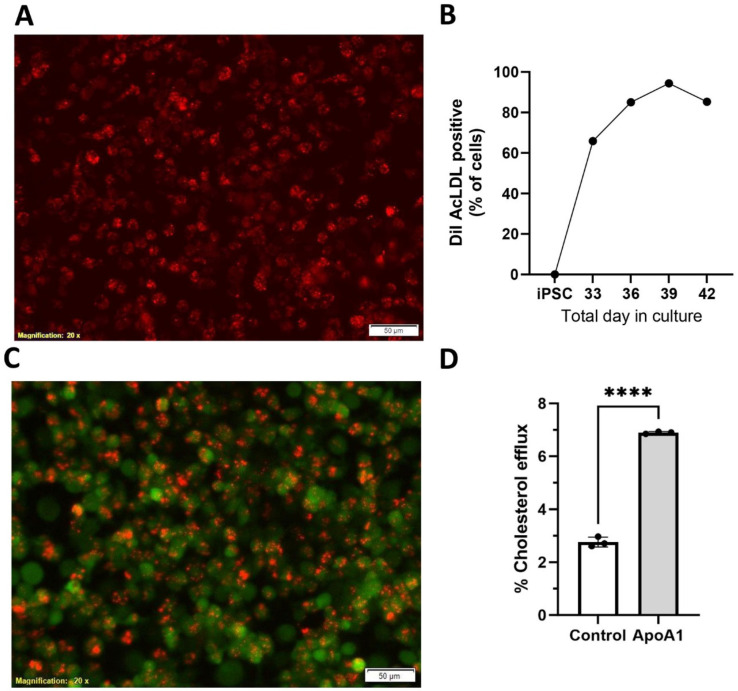
iPSMs display macrophage activities. (**A**) iPSMs uptake of DiI-labeled AcLDL, an assay for macrophage scavenger receptor-1. (**B**) % of DiI AcLDL loaded iPSMs by flow cytometry during phase 3 (4, 7, 10, and 13 days after day 29 progenitor harvesting). (**C**) Phagocytosis of pHrodo Red stained *E. coli* by iPSMs (membrane stained in green). (**D**) ApoA1-mediated cholesterol efflux from iPSMs. ****, *p* < 0.0001 by two-tailed *t*-test.

Macrophages express scavenger receptors and we assessed the ability of iPSMs to take up acetylated low-density lipoprotein (AcLDL), a non-physiological ligand for macrophage scavenger receptor 1, encoded by the MRS1 gene. iPSMs were incubated with DiI-labeled AcLDL for 25 min. At day 39, almost every iPSM took up the fluorescent AcLDL as observed by epifluorescent microscopy (Figure 5A). We used flow cytometry to quantify the % of DiI AcLDL positive iPSCs and iPSMs throughout phase 3. While iPSCs did not take up AcLDL, the % of iPSMs that took up AcLDL peaked at day 39 in culture, which was 10 days after starting phase 3 on day 29 (Figure 5B).

Macrophages are professional phagocytes and we assessed iPSM phagocytosis using pHrodo Red-labeled *E. coli*, which only is fluorescent in the red channel upon endosome acidification. All live cells were co-stained with a vital dye in the green channel. We observed robust phagocytosis by day 39 iPSMs, although not in every live cell (Figure 5C).

Human macrophages express ABCA1 constitutively [21] and can assemble nascent HDL leading to cholesterol efflux when exogenous apolipoprotein A1 (apoA1) is provided. Upon labeling iPSMs with [3H] cholesterol, we observed apoA1-mediated cholesterol efflux above baseline (Figure 5D).

Based on the time course, cell yields, and macrophage functional studies, we derived our optimal differentiation protocol, which involves 7 days of EB formation in phase 1, 29 days of total time (22 days in phase 2) for progenitor generation, and 39 days of total time (10 days after plating progenitors) for peak iPSM yield and maturation. We attempted to cryopreserve progenitors, but they were not viable after thawing, despite trying several freezing media. For optimal cryopreservation of iPSMs, we freeze the iPSMs at day 36 (7 days after plating progenitors). Upon thawing, the cells still have limited cell proliferation capacity, and we generally culture them for 3 days to yield additional cell numbers and complete their maturation.

### 3.5. Polarization of iPSMs

**Figure 6:**
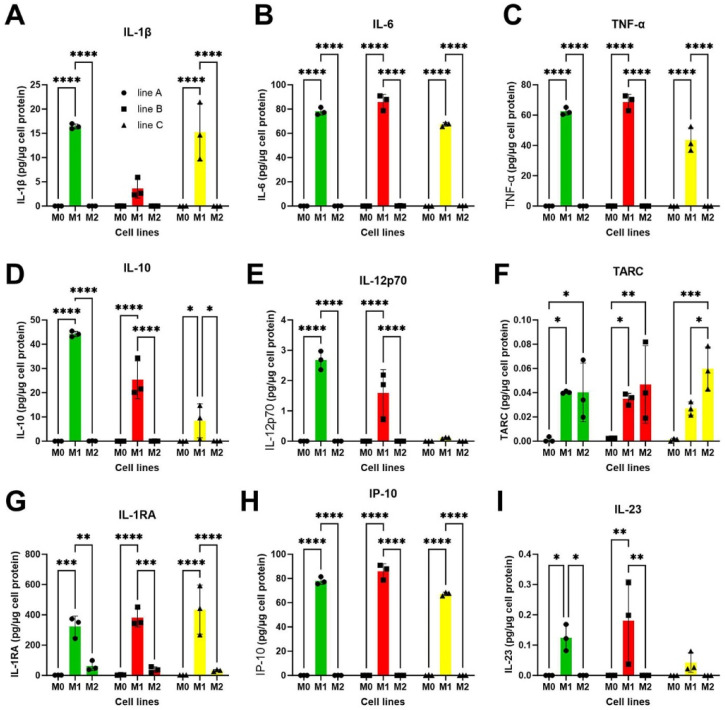
Cytokine release by iPSMs (M0) and polarized M1- and M2-like macrophages. Mature iPSMs (M0) of three iPSC lines (different symbols and colors) were treated with low-dose LPS (100 ng/mL) together with INFγ (20 ng/mL) for 24 h for the polarization to M1-like macrophages, or IL-4 (20 ng/mL) for 24 h for polarization to M2-like macrophages. The conditioned media of triplicate wells for each condition and cell line was collected for the assessment of multiple cytokines. (**A**–**I**) IL-1β, IL-6, TNFα, IL-10, IL-12p70, TARC, IL-1RA, IP-10, and IL-23 levels, respectively, released by M0, M1-and M2-like macrophages. * *p* < 0.05, ** *p* < 0.01, *** *p* < 0.001, **** *p* < 0.0001 by two-tailed ANOVA with Tukey’s posttest.

To investigate the effects of polarization on iPSMs, mature iPSMs derived from three iPS donors (A–C) on day 39 were subjected to 24-h treatments to polarize them into M1-like macrophages, using a low dose of lipopolysaccharide (LPS) combined with interferon-gamma (IFNγ), or into M2-like macrophages, using interleukin-4 (IL-4). Post-treatment, cell supernatants were analyzed using a multiplex cytokine assay panel to assess secretion of IL-1β, IL-6, TNFα, IL-10, IL-12p70, TARC, IL-1RA, IP-10, and IL-23 (Figure 6A–I). The cytokine analysis revealed distinct secretion profiles characteristic of M0 (non-polarized), M1-like, and M2-like macrophages. Notably, M1-like macrophages exhibited significantly higher levels of cytokine secretion compared to M0 (non-polarized) and M2-like macrophages across most measured cytokines. However, both M1-like and M2-like macrophages vs. M0 showed high secretion levels of TARC (Figure 6F). Variations in cytokine secretion patterns were observed among different cell line donors, suggesting potential genetic influences on secretion outcomes after polarization.

**Figure 7:**
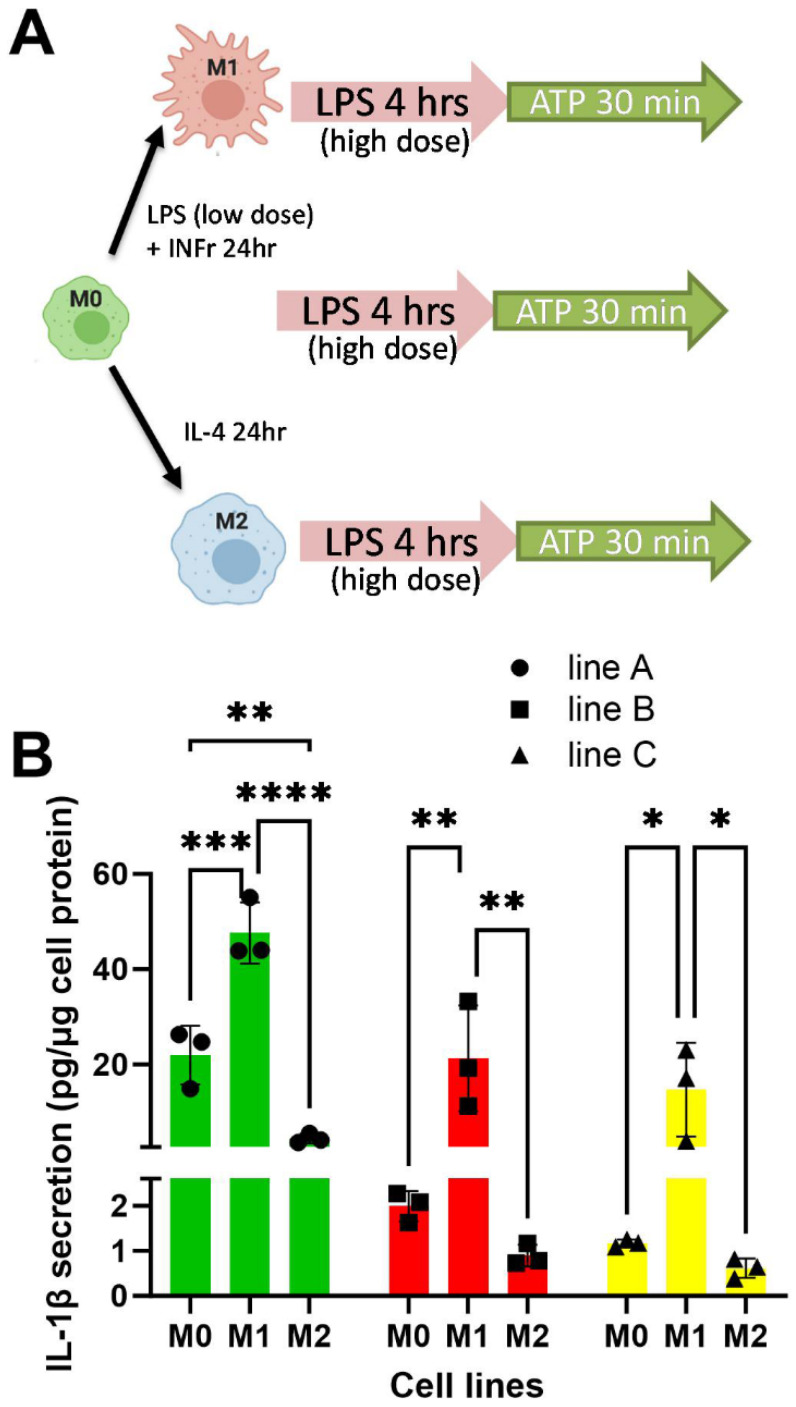
Inflammasome mediated IL-1β release from M0, M1-like, and M2-like iPSMs derived from three iPS lines (different symbols and colors). (**A**) Schematic of iPSM polarization and inflammasome priming with high dose LPS (1 μg/mL for 4 h followed by activation with ATP (5 m M) for 30 min. (**B**) IL-1β levels in the conditioned media (normalized to cellular protein mass) in triplicate wells of M0, M1- and M2-like iPSMs from each cell line. * *p* < 0.05, ** *p* < 0.01, *** *p* < 0.001, and **** *p* < 0.0001 by two-tailed ANOVA with Tukey’s posttest.

The M0, M1-like, and M2-like macrophages were also subjected to acute inflammasome priming with a high dose of LPS followed by inflammasome activation with exogenous ATP treatment (Figure 7A), and secreted IL-1β levels were assessed in the media. Although inflammasome-mediated IL-1β was detected in all three macrophage states, and from all donor cell lines, IL-1β levels were highest from M1-like macrophages, with lower levels released from M0 and M2-like macrophages (Figure 7B). For iPSMs derived from donor A, M0 macrophages released more IL-1β than M2-like cells, and a similar trend was observed from donors B and C (Figure 7B). Again, potential donor genetic differences might account for the varied inflammasome responses.

### 3.6. RNAseq Analysis

**Figure 8:**
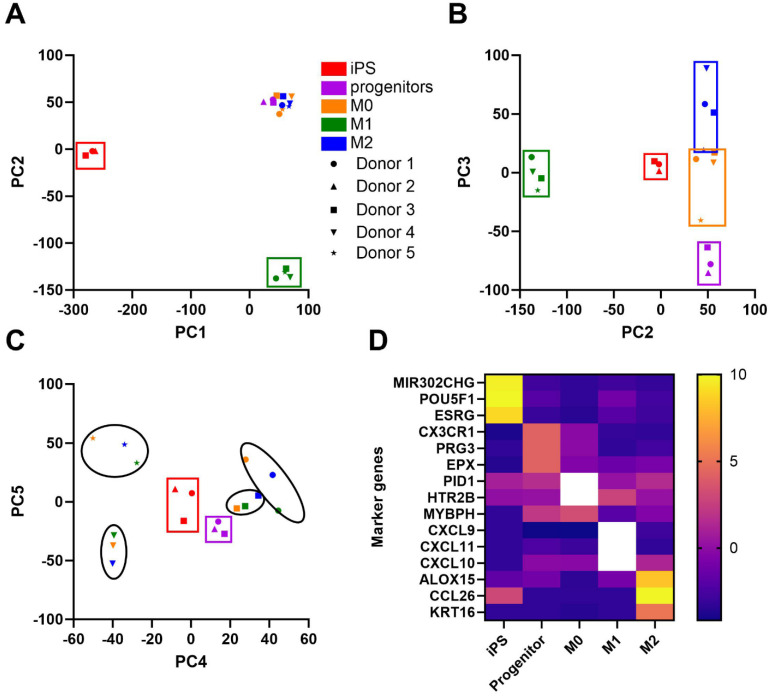
RNAseq of iPSCs, macrophage progenitors, and iPSMs from different iPS donors. (**A**–**C**) Principal component analysis for the first 5 principal components (PCs). Different cell types are shown in different colors and different donors are depicted by different shaped symbols. Colored rectangular frames represent clustering by cell type, and black oval frames represent a clustering of M0, M1- and M2-like iPSMs by the donor. (**D**) Marker genes for the 5 cell types were identified from RNAseq based on log2 fold change for the selected cell type vs. the other cell types. The color bar shows log2 counts per million, with values >10 shown in white.

iPSCs and macrophage progenitors from 3 different donors and M0, M1-like, and M2-like macrophages from 4 different donors were prepared and subjected to RNAseq. We used principal component (PC) analysis to separate the samples into clusters. PC1 distinguished iPSCs from all other cell types and PC2 distinguished M1-like and iPSCs from the remaining cell types (Figure 8A, cell types displayed by different colors within rectangular frames). PC2 combined with PC3 distinguished all cell types fairly well, with a small overlap between M0 and M2-like macrophages (Figure 8B). The donors for M0, M1, and M2-like macrophages were clearly distinguished by PC4 combined with PC5, likely representing the effects of common genetic variation on gene expression in macrophages (Figure 8C, cell donors displayed by different symbols within oval frames). PCs 4 and 5 also distinguished the iPSCs and progenitors from the other cell types, regardless of their donors. Marker genes were selected that best distinguished between the various cell types based on their differential gene expression, selecting only for protein-coding or microRNA-coding genes (Figure 8D). The top maker genes for iPSCs included *mir302CHG*, *POU5F1* (encoding the Yamanaka factor OCT4) [4], and *ESRG* (embryonic stem cell-related gene). Other well-known iPS genes, such as *NANOG* and *SOX2*, were also enriched vs. the other cell types (log2 fold change vs. all other types = 9.72 and 5.04, respectively). The macrophage progenitor top marker genes were *CX3CR1* (fractalkine receptor), *PRG3* (proteoglycan 3), and *EPX* (eosinophil peroxidase). The M0 marker genes were *PID1* (phosphotyrosine interaction domain-containing protein 1), *HTR2B* (5-hydroxytryptamine receptor 2B), and *MYBPH* (myosin binding protein H; although this marker was also expressed in progenitors). The M1 maker genes were all chemokines, *CXCL9*, *CXCL11*, and *CXCL10* (IP-10), the latter of which was shown at the protein level to be enriched in M1-like cells (Figure 6H). The M2 maker genes were *ALOX15* (arachidonate 15-lipoxygenase), the chemokine *CCL26*, and *KRT16* (keratin 16).

## 4. Discussion

Both monolayer and EB-based methods have been used for the differentiation of iPSCs to macrophages [6,8,9,22,23,24]. The monolayer method is complex, involving multiple different growth factors and cytokines used at different days during the differentiation [9,22,23,24]. In our hands, we failed to obtain reproducible macrophages following the monolayer method by Shi et al. [22]. The prior EB-based methods used low-adherence plates for the formation of EBs from iPSCs [6,8,9]. In our hands, forming EBs on low adherence plates by the method of Nenashiva et al. [8] yielded EBs that aggregated forming EBs of variable sizes that upon transfer to gelatin-coated plates led to decreased adherence and decreased release of iPSM progenitors. This inconsistency led to a low success rate, with over half of our attempts failing with this method. Another EB method used low adhesion 96 well plates to form one EB in each well [6]. Although these EBs were more uniform in size, yields of macrophages were limited in our hands. This study also mentions the use of AggreWell plates, but no details of the conditions used were specified [6]. One study compared the monolayer and EB methods, which concluded that the monolayer method was more reproducible [9].

In the current study, we integrated several features to develop an optimized and simplified EB-based method for the differentiation of iPSMs, which reproducibly yielded functioning macrophages. The key innovations include: (1) standardized EB formation using AggreWell plates in phase 1 to ensure uniform EB size; (2) cell filtration during progenitor harvesting to remove EB fragments and cell aggregates; (3) optimized culture conditions with a low oxygen environment at the start of phase 2 to improve EB adherence and growth; (4) strict iPSC quality control by limiting iPSCs to no more than 50% confluence before starting phase 1 to enhance differentiation outcomes; and (5) performing a detailed time course throughout phases 2 and 3 to optimize the quality and quantity of iPSMs. These methodological enhancements resulted in high consistency and success across multiple iPSC cell lines.

The use of AggreWell plates in Phase 1 of our revised method ensured the controlled formation and growth of each EB. This approach enabled us to standardize EB formation and synchronize the release of macrophage progenitors in the subsequent phase, enhancing the reproducibility and reliability of the process. When harvesting floating macrophage progenitors at the end of phase 2, we observed floating cell aggregates composed of EB fragments. The inclusion of these secondary EBs in phase 3 prevented the uniform maturation of iPSMs as new progenitors were being released. To address this, we implemented cell filtration during progenitor harvesting to remove these secondary EBs, thereby preserving the homogeneity of the progenitors moved to phase 3 for iPSM maturation.

A few advances in our method were made empirically via observations after trying alternate modifications in pilot studies. We discovered that culturing EBs in a low oxygen environment at the start of phase 2 enhanced the yield of progenitors. Additionally, we found that the confluency of iPSCs prior to differentiation played a key role in successful macrophage differentiation. We evaluated iPSC confluences of 25%, 50–60%, and 75–90% at the initiation of differentiation and found that 50% confluent was the most effective. Starting the differentiation with iPSCs at lower confluence required more plates of cells to make the EBs while starting with iPSCs at 75–90% confluence led to the formation of EBs in Phase 1, which were less compact, fragile, and difficult to keep intact while pipetting off the AggreWell plate.

We also looked at the time course during phases 2 and 3. Previous research suggested that extending the production of macrophage progenitors to eight weeks could increase the yield of macrophages, with progenitor production potentially continuing for up to a year [6]. However, we observed that the quantity of progenitors decreased from EBs after day 35 (Figure 4B), and the quality of the progenitors may be compromised as shown by decreased CD14 expression (Figure 4A). We found that limiting the harvesting of progenitors from day 29 to day 42 yielded good quality and quantity of iPSMs while harvesting progenitors on day 29 followed by a 10-day iPSM maturation yielded the optimal outcome. This single harvest day maximized yield while minimizing both time and resources. Notably, if phase 3 iPSM maturation was lengthened beyond day 13 we observed the emergence of fibroblast-like cells that began to outnumber and displace the iPSMs.

In addition to providing an optimized protocol for iPSM production, our characterization of the differentiation state and iPSM polarization includes cytokine release and transcriptome analysis via RNAseq for iPS cells, macrophage progenitors, M0, M1-, and M2-like macrophages. Most cytokines were released higher by M1-like macrophages than either M0 or M2-like macrophages, including IL-10, which is usually associated with M-2-like, rather than M1-like, macrophages. The RNAseq data revealed that IL-10 mRNA was expressed in M0, M1-like, and M2-like macrophages. Thus, we assume that intracellular levels of IL-10 would be found in M2-like macrophages, but release of IL-10 into the media was not observed, as a secretory signal was not present in our 1-day M2-like polarized cells. We found a similar result where M2-like macrophages derived from human peripheral blood monocytes did not secrete IL-10 unless a second stimulus was added [25].

The analyzed RNAseq data, available via GEO accession # GSE277609, was subjected to PC analysis, which demonstrated excellent discrimination among these cell states (PCs 1–3) and also for the cell donor (PCs 4–5, but only for the M0, M1-, and M2-like macrophages). The transcriptome PC analysis tight clustering by cell type and donor demonstrates the reproducibility of our method as well as the strong genetic basis for regulating gene expression, as discovered in eQTL studies, such as those identified in mouse bone marrow macrophages [26] and human tissues in the GTEx Project [27]. In the future, we plan to leverage~100 human iPS lines from control subjects to perform GWAS in a dish [28] for various macrophage phenotypes as well as eQTL studies (transcriptome combined with SNP microarrays) to identify common human regulatory variants associated with the expression of macrophage genes. For example, we can subject the iPSM to LPS and other stimuli and discover genetic variants associated with innate immunity and inflammation and its resolution, which would not be possible in living human subjects. A similar study has previously been performed in human monocyte-derived dendritic cells [29], however, primary monocytes are a limited resource, while iPS cells are practically unlimited in their ability to proliferate, and thus may be used again and again for different studies and to show reproducibility. These iPSM studies may shed light on the GWAS loci identified for many diseases that involve innate immunity, such as atherosclerosis and inflammatory bowel disease. In addition, using CRISPR-Cas9 gene editing in iPSCs to create disease-causing mutations, we could model macrophage phenotypes in iPSMs.

## 5. Conclusions

We successfully developed a novel simplified and efficient three-phase iPSM differentiation method, for which we have meticulously monitored both the quantity and characteristics, including transcriptomes, of cells at each phase. Our method has proven reproducible with different iPS donors. The iPSMs we created retain classical macrophage functions, validating their potential use for various biomedical applications.

## Figures and Tables

**Table 1:** EB medium (for phase 1).

Composition	Volume (for 50 mL)	Final Concentration
mTeSR medium	50 mL	
BMP-4 (25 μg/mL stock) ^1^	100 μL	50 ng/mL
SCF (100 μg/mL stock) ^2^	10 μL	20 ng/mL
VEGF (50 μg/mL stock) ^3^	50 μL	50 ng/mL

^1^ BMP-4 (R&D Systems, Minneapolis, MN, USA # 314-BP-050). ^2^ SCF (R&D Systems, Minneapolis, MN, USA # 255-SC-010/CF). ^3^ VEGF (R&D Systems, Minneapolis, MN, USA # 293-VE-500).

**Table 2:** Progenitor culture medium for phase 2, adapted from [8].

Composition	Volume (for 50 mL)	Final Concentration
X-VIVO-15	50 mL	
M-CSF (100 μg/mL stock) ^1^	50 μL	100 ng/mL
IL-3 (100 μg/mL stock) ^2^	25 μL	50 ng/mL
Glutamax (200 mM stock)	500 μL	2 mM
2-Mercaptoethanol (55 mM)	90 μL	0.1 mM
Penicillin/Streptomycin (10,000 units/mL)	500 μL	100 units/mL

^1^ M-CSF (BioLegend, San Diego, CA, USA # 574808) ^2^ IL-3 (GeneScript, Piscataway, NJ, USA # Z03156).

**Table 3:** iPSM maintenance medium (for phase 3).

Composition	Volume (for 10 mL)	Final Concentration
RPMI1640	8 mL	
Penicillin/Streptomycin (10,000 units/mL)	100 μL	100 units/mL
FBS	2 mL	20%
M-CSF (100 μg/mL stock)	10 μL	100 ng/mL

**Table 4:** Antibody reagents.

Antigen	Fluorochrome	Clone	BD ^1^ Catalog#
CD45	BB515	HI30	564585
CD86	BB700	2331(FUN-1)	566473
CD14	APC H7	MoP-9	560180
CD163	BV650	GHI/61	563888
CD11c	PE	BU15	566730
CD90	PECy7	5E10	561558

^1^ BD Biosciences (Franklin Lakes, NJ, USA).

## Data Availability

The RNAseq DESeq2 normalized CPM are available via Gene Expression Omnibus (GEO) accession # GSE277609. Other data are available from the corresponding authors upon reasonable request.

## Supplementary Materials

The following supporting information can be downloaded at: https://www.mdpi.com/article/10.3390/biomedicines13010099/s1, Figure S1: Scatter plots and gating of forward and side scatter for cell lines shown in Figure 3.

## Abbreviations

The following abbreviations are used in this manuscript:

iPSC	induced pluripotent stem cell
EB	embryoid body
iPSMs	iPSC-derived macrophages
FC	flow cytometry
AcLDL	Aceteylated LDL
ATP	adenosine triphosphate
BCA	bicinchoninic acid
CPM	counts per million
PCA	principal component analysis
GEO	Gene Expression Omnibus
apoA1	apolipoprotein A1
LPS	lipopolysaccharide
IFNγ	Interferon gamma
IL-4	interleukin-4
