# Supplementary material for: Optimized Method to Generate Well-Characterized Macrophages from Induced Pluripotent Stem Cells
Source: Biomedicines. 2025 Jan 3;13(1):99. doi: 10.3390/biomedicines13010099 (PMC11762477; doi:10.3390/biomedicines13010099)
Supplement: Supplementary file 1 [file biomedicines-13-00099-s001.zip › biomedicines-3412970-supplementary.pdf]

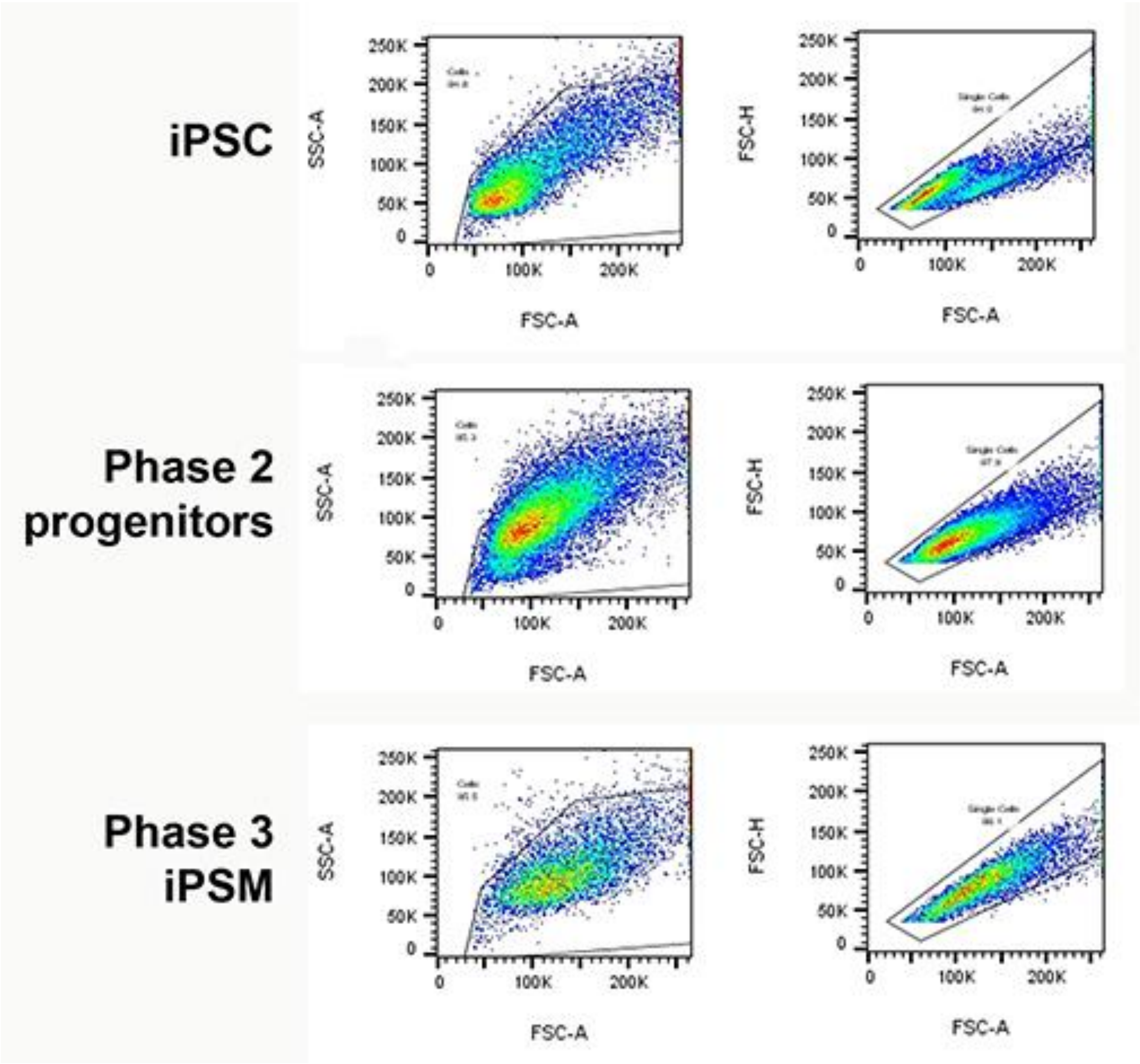


**Supplemental Figure S1**. Scatter plots and gating of forward and side scatter for cell lines shown in Figure 3
